# Design of a large-scale femtoliter droplet array for single-cell analysis of drug-tolerant and drug-resistant bacteria

**DOI:** 10.3389/fmicb.2013.00300

**Published:** 2013-10-04

**Authors:** Ryota Iino, Yoshimi Matsumoto, Kunihiko Nishino, Akihito Yamaguchi, Hiroyuki Noji

**Affiliations:** ^1^Department of Applied Chemistry, Graduate School of Engineering, The University of TokyoTokyo, Japan; ^2^Core Research for Evolutional Science and Technology, Japan Science and Technology AgencyTokyo, Japan; ^3^Laboratory of Microbiology and Infectious Diseases, Institute of Scientific and Industrial Research, Osaka UniversityOsaka, Japan; ^4^Department of Cell Membrane Biology, Institute of Scientific and Industrial Research, Osaka UniversityOsaka, Japan; ^5^Graduate School of Pharmaceutical Sciences, Osaka UniversityOsaka, Japan

**Keywords:** single-cell analysis, microdevice, drug tolerance, persister, drug resistance, drug efflux, transporter

## Abstract

Single-cell analysis is a powerful method to assess the heterogeneity among individual cells, enabling the identification of very rare cells with properties that differ from those of the majority. In this Methods Article, we describe the use of a large-scale femtoliter droplet array to enclose, isolate, and analyze individual bacterial cells. As a first example, we describe the single-cell detection of drug-tolerant persisters of *Pseudomonas aeruginosa* treated with the antibiotic carbenicillin. As a second example, this method was applied to the single-cell evaluation of drug efflux activity, which causes acquired antibiotic resistance of bacteria. The activity of the MexAB-OprM multidrug efflux pump system from *Pseudomonas aeruginosa* was expressed in *Escherichia coli* and the effect of an inhibitor D13-9001 were assessed at the single cell level.

## INTRODUCTION

Opportunistic infection with bacteria resistant to multiple antibiotics is a continuing clinical challenge (Taubes, 2008). The antibiotic resistance of bacteria can be classified into two categories, natural resistance (tolerance) and acquired resistance. In natural resistance, a very small proportion of the bacterial population is resistant to multiple antibiotics despite having the same genotype as the sensitive majority. These bacteria are often referred to as “persisters” (Lewis, 2010). However, the nature of these persisters is not fully understood because they occur at a very low frequency in a bacterial population (typically less than 1%), which makes systematic studies difficult (Allison et al., 2011; Balaban, 2011, Gerdes and Maisonneuve, 2012; Kint et al., 2012). In the first section of this review article, we describe a microdroplet-based method to identify and culture individual bacterial cells for efficient detection of persisters. In contrast, acquired antibiotic resistance is caused by a change in the genotype of the sensitive strain. The four main mechanisms of acquired resistance are suppression of drug influx into the cell due to decreased expression of membrane channel proteins, inactivation of drugs by intracellular and extracellular enzymes, mutations in the target proteins of drugs, and active efflux of the drugs from the cell due to increased expression of efflux pumps (Fischbach and Walsh, 2009; Nikaido, 2009). Here we focused on the active efflux of drugs from the cell. In the second section, we introduce a microdroplet-based method for assessing the drug efflux activity of single bacterial cells.

## ADVANTAGES OF SINGLE-CELL ANALYSIS USING A MICRODEVICE

Single-cell analysis is a powerful approach for detecting variations among the cells in a population, such as differences in the expression of proteins, the copy number of genes, and the concentration of metabolites (Li and Xie, 2011; Trouillon et al., 2013). Single-cell analysis can overcome the limitations associated with ensemble-averaged data from multiple cells, and enable the identification of very rare cells with properties that differ from those of the majority. Microfabricated devices have contributed greatly to the development of massively parallel and high-throughput single-cell analyses.

However, in most microdevices, the target cells are eukaryotic, such as mammalian cells and yeasts (Sims and Allbritton, 2007; Gupta et al., 2010, Lindstrom and Andersson-Svahn, 2010), because their size, a few millimeters to tens of micrometers, allows for easy handling compared to bacteria, which are much smaller in size. Thus far, only a few studies have used microdevices for single bacterial cell analysis (Balaban et al., 2004; Cai et al., 2006, Ottesen et al., 2006; Weibel et al., 2007, Boedicker et al., 2009; Teng et al., 2013, Wakamoto et al., 2013). In addition, in many microdevices that are based on microfluidic channels and valves or droplets generated in a microfluidic channel, the closed nature of the system makes the collection of cells from the device and their subsequent use difficult. Therefore, the development of microdevices from which individual bacterial cells can be recovered has been highly anticipated.

## LARGE-SCALE FEMTOLITER DROPLET ARRAY FOR SINGLE BACTERIAL CELL ANALYSIS

We recently developed a micron-sized femtoliter droplet array fixed on a hydrophilic-in-hydrophobic micropatterned surface (Sakakihara et al., 2010). In our new microdevice, a large number of dome-shaped femtoliter droplets can be prepared that enclose individual bacterial cells (Iino et al., 2012a). One prominent feature of this array is that the individual droplets containing the enclosed cells can be accessed and collected with a micropipette. The array can also be used for mass culture and gene and protein analyses.

**FIGURE 1:**
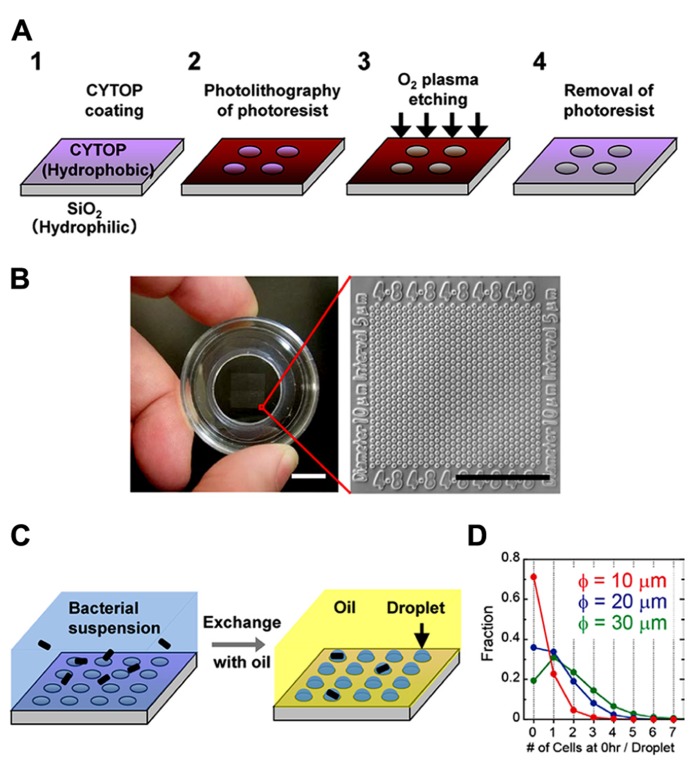
**Formation of a femtoliter droplet array containing bacteria. (A)** Schematic of the fabrication procedure for preparation of a hydrophilic-in-hydrophobic micropatterned surface. **(B)** Image of the assembled device (left). Scale bar, 10 mm. Microscopic image of the hydrophilic-in-hydrophobic micropatterned surface (right). Scale bar, 200 μ m. **(C)** Procedure for bacterial enclosure into the femtoliter droplet array. **(D)** The number of bacterial cells in each droplet in microdevices with different hydrophilic surface diameters just after enclosure.

We prepared the hydrophilic-in-hydrophobic micropatterned surface through conventional microfabrication (**Figure 1A**). A hydrophobic polymer of carbon-fluorine (CYTOP; Asahi Glass, Japan) was deposited on a SiO_2_ cover glass, and photolithography was performed using a high-viscosity photoresist. The resist-patterned substrate surface was dry-etched with O_2_ plasma by using a reactive ion-etching system to produce circular micropatterns on a hydrophilic SiO_2_ glass surface. The diameter of the exposed hydrophilic SiO_2_ surfaces was 10–30 μ m, and they were surrounded by a hydrophobic polymer layer with a height of 1 μ m. A fabricated micropatterned cover glass was attached to the bottom of a perforated petri dish (**Figure 1B**). The circular micropatterns were grouped into islands and were numbered to identify the individual droplets and the cells enclosed in each droplet.

To form a droplet array containing bacteria, we covered the micropatterned cover glass with medium containing a bacterial suspension (**Figure 1C**, left). Then, fluorinated oil (Fluorinert FC-40; Sigma Aldrich, USA), which has a higher density than water, was flowed over the medium near the surface. The hydrophilic SiO_2_ glass surfaces retained the medium and the bacteria, whereas the hydrophobic surface was replaced with oil. As a result, many droplets containing one or more bacteria were formed (**Figure 1C**, right). More than 3 × 10^5^ droplets could be simultaneously prepared in a 1-cm^2^ area in a single device. Enclosure of the cells in the droplets was stochastic and was dependent on the cell density of the bacterial suspension. At an optical density (turbidity) at 600 nm (OD_600_) of 0.6, approximately 20–30% of the droplets contained single cells. Increasing the diameter of the hydrophilic surfaces to 20 or 30 μ m increased the fraction of droplets containing multiple cells; however, the fraction of droplets containing single cells did not increase significantly (**Figure 1D**). In contrast, the number of droplets formed increased significantly when we used a device with hydrophilic surfaces of a smaller diameter. Therefore, to increase the total number of droplets containing single cells, we used a microdevice containing hydrophilic surfaces with a diameter of 10 μ m.

## DETECTION OF PERSISTER BACTERIA IN A FEMTOLITER DROPLET ARRAY

**FIGURE 2:**
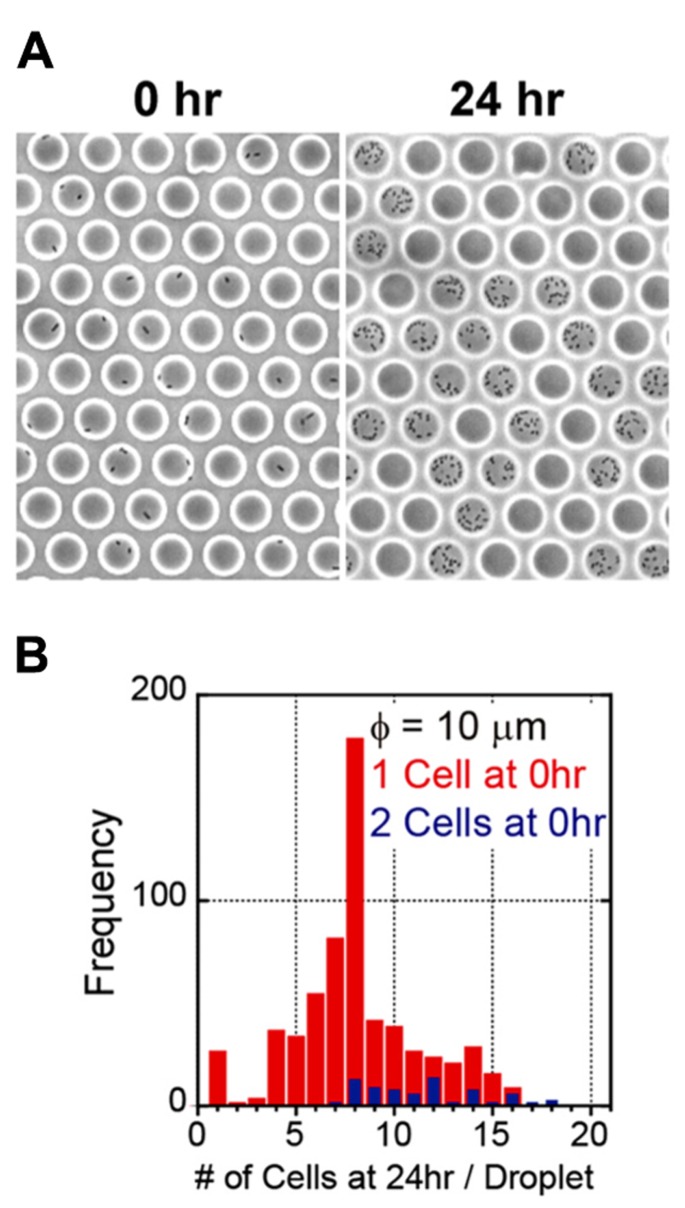
**Enclosure and culture of bacteria in a femtoliter droplet array. (A)** Images of *P. aeruginosa* PAO1 after 0 h (left) and 24 h (right) of culture in the femtoliter droplet array. **(B)** Distribution of the number of cells after 24 h of culture.

**FIGURE 3:**
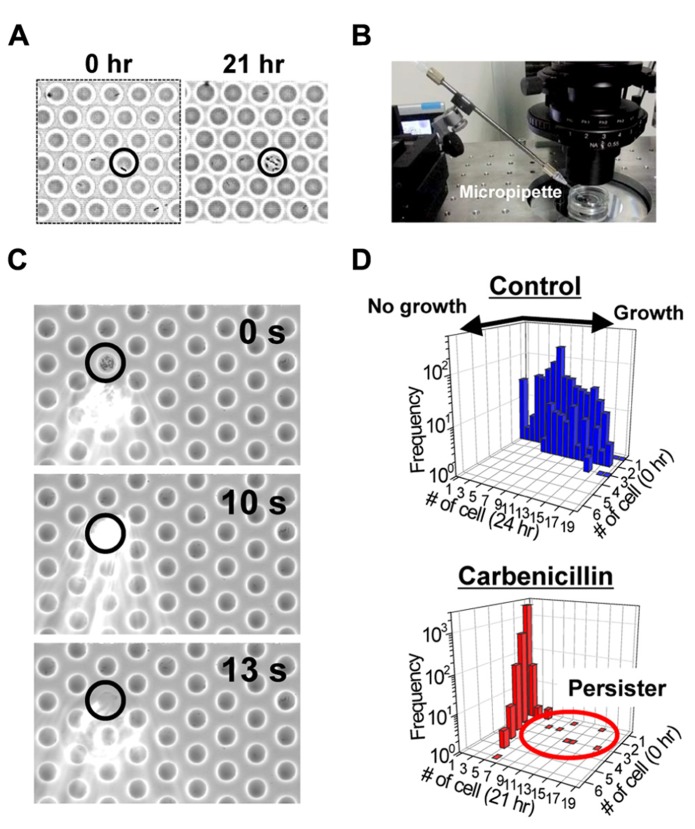
**Detection of the persisters in the femtoliter droplet array. (A)** Images of *P. aeruginosa* PAO1 persisters (indicated by circle) after 0 h (left) and 21 h (right) of culture in the femtoliter droplet array. **(B)** Image of the micropipette used for droplet collection. **(C)** Sequential images of droplet collection. **(D)** Distribution of the number of cells in each droplet before and after overnight culture. Top, control cells without carbenicillin treatment. Bottom, carbenicillin-treated cells.

We generated a femtoliter droplet array of *Pseudomonas aeruginosa* PAO1 using our microdevice to detect persisters under an optical microscope. In the control experiment without antibiotic treatment, most cells underwent multiple cell divisions after incubation overnight at 37°C (**Figures 2A,B**). The divided cells showed active flagellar motion, indicative of high metabolic activity. To detect persisters, an antibiotic, carbenicillin (at final concentration of 5 mg/mL, which is ~100 times higher than the minimal inhibitory concentration), was added to the bacterial suspension that was grown to late exponential phase (OD_600_ ~1.0) in trypticase soy broth. The suspension was further incubated at 37°C for 3 h, and then the cells were collected, washed, resuspended in fresh medium (OD_600_ ~0.2). This suspension was enclosed in a droplet array. After enclosure, the whole device was placed in an incubator at 37°C, and the cells were cultured overnight. The persisters were easily identified under an optical microscope the overnight culture (**Figure 3A**). The divided cells were not cells that acquired resistance, but were actually persisters. This was confirmed by collecting the cells with a micropipette with an aperture diameter of 10–15 μ m (**Figures 3B,C**), inoculating a culture in test tubes, and antibiotic susceptibility testing.

Bacterial cells that divided multiple times were counted, and the frequency of persisters was calculated. The frequency of persisters in the femtoliter droplet array (1.5 ± 0.72%, *N* = 4; **Figure 3D**) was quite unexpectedly much higher than that estimated by conventional agar plate assays (0.10 ± 0.03%, *N* = 4). In the plate assays, the carbenicillin-treated preculture sample was prepared as described above along with an untreated culture sample, and then the samples were serially diluted and cultured overnight at 37°C on agar plates. The number of colonies on the plates from carbenicillin-treated and untreated preculture samples were counted and compared. It has been recently reported that quorum sensing autoinducer increased the frequency of persister appearance (Moker et al., 2010; Leung and Levesque, 2012, Vega et al., 2012), and that inhibiting the quorum signal restored antibiotic susceptibility (Pan et al., 2012, 2013). Furthermore, the quorum-sensing signal could be transduced even in single isolated cells when PAO1 was enclosed in picoliter-volume droplets (Boedicker et al., 2009). Therefore, enclosure of a single cell in a femtoliter droplet may enhance the quorum sensing signal and increase persister frequency. The effect of the quorum sensing signal on the frequency of persister appearance in the femtoliter droplet array can be more clearly confirmed by treating the cells with antibiotic after enclosure in the droplets by adding the antibiotic with a micropipette (Sakakihara et al., 2010).

## A SINGLE-CELL DRUG EFFLUX ASSAY IN A FEMTOLITER DROPLET ARRAY

The AcrAB-TolC multicomponent efflux pump system recognizes and expels a wide variety of compounds, including antibiotics, dyes, and detergents. In this system, AcrA is the membrane fusion protein that stabilizes the complex (Zgurskaya and Nikaido, 1999), AcrB is the inner membrane transporter protein that belongs to the resistance-nodulation-division (RND) family (Murakami et al., 2006; Nakashima et al., 2011, 2013), and TolC is the outer membrane channel protein (Koronakis et al., 2000). The AcrAB-TolC efflux system is responsible for both intrinsic and acquired drug resistance of Gram-negative bacteria such as *Escherichia coli* and *Salmonella enterica* (Nishino and Yamaguchi, 2008; Nikaido and Takatsuka, 2009). Two systems *P. aeruginosa *that are homologous to the AcrAB-TolC system, MexAB-OprM and MexXY-OprM, lead to multidrug resistance in clinical isolates (Morita et al., 2001; Livermore, 2002; Hocquet et al., 2006, 2007; Henrichfreise et al., 2007).

**FIGURE 4:**
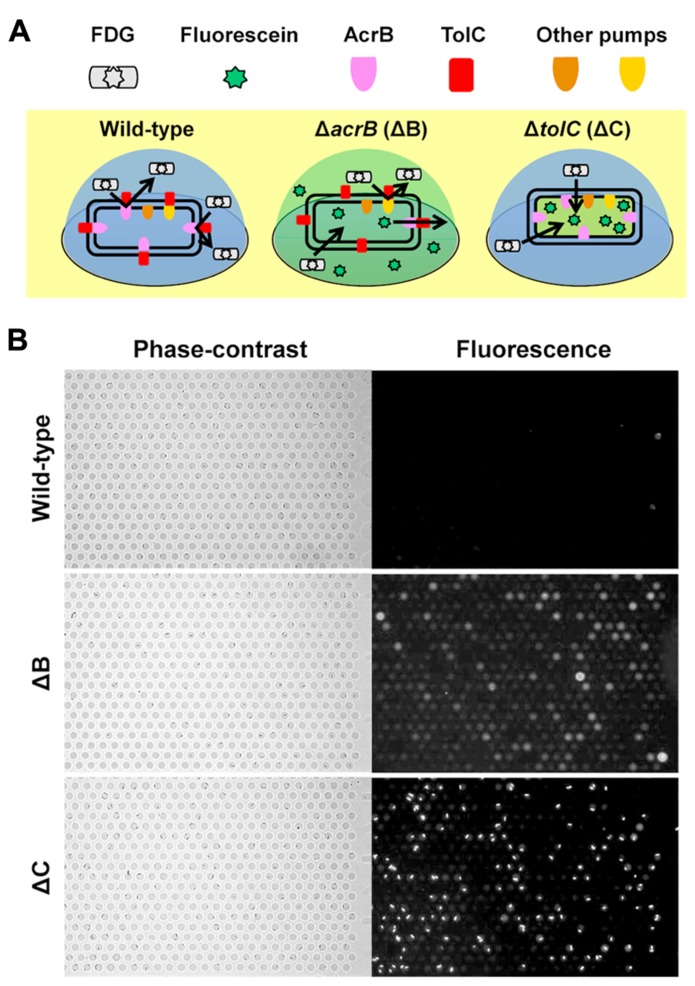
**Single-cell drug efflux assay. (A)** Schematic of the principle of the single-cell drug efflux assay. **(B)** A representative assay. Phase-contrast (left) and fluorescence (right) images of the same field are shown.

We have recently developed a single-cell drug efflux assay using the femtoliter droplet array (**Figure 4**; Iino et al., 2012a). In this assay, *E. coli* cultured in test tubes was mixed with a fluorogenic substrate, fluorescein-di-β -D-galactopyranoside (FDG), enclosed in a droplet array, and then cultured for 15–20 min at room temperature. Upon entering the cytoplasm of *E. coli*, FDG is hydrolyzed into the fluorescent dye fluorescein by β -galactosidase. Both FDG and fluorescein are substrates for the AcrAB-TolC system (Matsumoto et al., 2011; Iino et al., 2012b). In wild-type cells, FDG was effectively pumped out before hydrolysis, and no fluorescence was detected (**Figure 4A**, left, and **Figure 4B**, top). In contrast, when FDG was imported intoΔ *acrB* (Δ B) andΔ *tolC* (Δ C) strains it was hydrolyzed to fluorescein. InΔ B cells, not only the cells, but also the droplets themselves fluoresced (**Figure 4A**, center, and **Figure 4B**, middle) because the remaining minor RND efflux pumps slowly pumped out the fluorescein. Although only a small amount of the dye was pumped out, it could be easily detected because it was confined to the femtoliter droplet (, Kim et al., 2012). InΔ C cells, fluorescein accumulated in the cell (**Figure 4A**, right, and **Figure 4B**, bottom) because TolC is a channel protein common to both the major and minor RND efflux pumps in *E. coli*.

**FIGURE 5:**
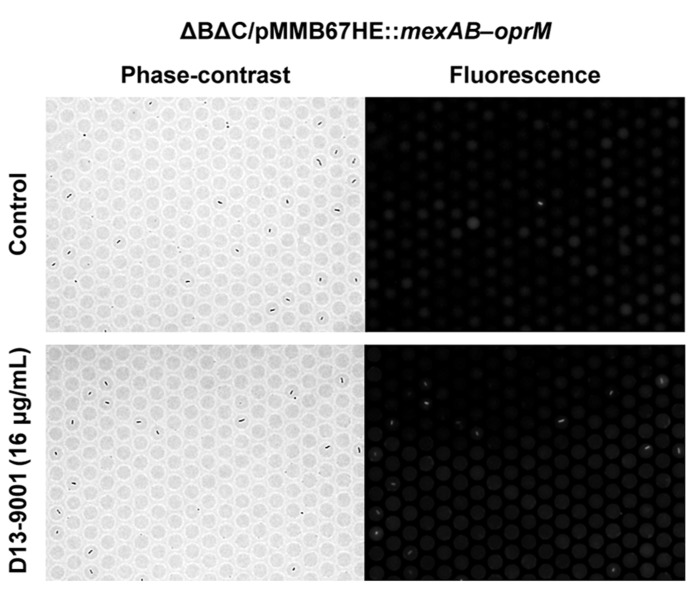
**Effect of the pump inhibitor D13-9001 on the efflux activity of MexAB-OprM expressed in theΔ BΔ C *E. coli* strain.** Phase-contrast (left) and fluorescence (right) images of untreated control cells (top) or cells treated with 16 μ g/mL D13-9001 (bottom).

With this method, the inhibitory effect of chemical compounds against the efflux pump can be easily assessed. The effect of an efflux pump inhibitor, D13-9001 (Yoshida et al., 2007), is shown in **Figure 5**. D13-9001 has been reported to enhance the antibacterial activities of several antibiotics by binding tightly to the drug binding pockets of AcrB and MexB (Nakashima et al., 2013). AΔ BΔ C double-knockout *E. coli* strain that stably expresses MexAB-OprM from *P. aeruginosa* was used for the experiment. This strain did not fluoresce in our assay, indicating that the exogenously expressed MexAB-OprM worked well in *E. coli*, and that the cells recovered drug efflux activity (**Figure 5**, top). Addition of D13-9001 increased the number of fluorescent cells (**Figure 5**, bottom). The fluorescence intensity of the cells increased as the concentration of D13-9001 increased, indicating a concentration-dependent inhibitory effect. D13-9001 is a specific inhibitor of MexB, a major efflux pump in *P. aeruginosa*. However, it does not inhibit MexY, which is another major efflux pump in *P. aeruginosa *(Yoshida et al., 2007). Our simple and rapid approach would be useful to screen for new inhibitors that are also effective against MexY and other efflux pumps.

## PHENOTYPIC CHANGE AFTER GENETIC TRANSFORMATION

**FIGURE 6:**
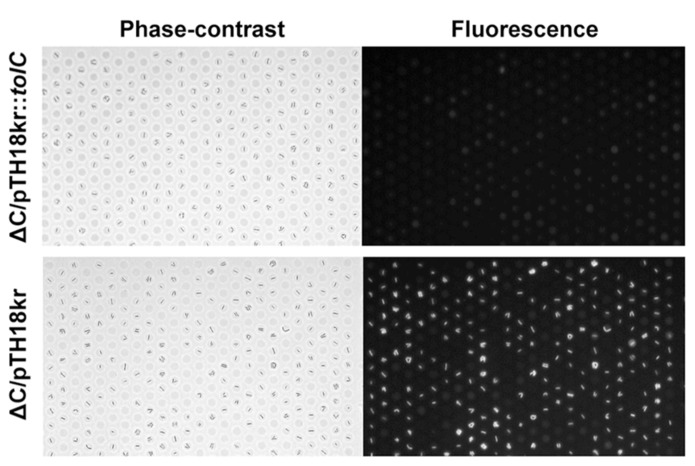
**Phenotypic change after genetic transformation.** Phase-contrast (left) and fluorescence (right) images of *E. coli*Δ C cells transformed with a vector expressing *tolC *from *Salmonella enterica *(pTH18kr::tolC; top) or a control vector pTH18kr (bottom).

As a demonstration of the rapid phenotypic change after genetic transformation, we introduced the *S. enterica* *tolC* gene into *E. coli*Δ C cells. After electroporation with the expression vector, the cells were incubated for different time durations in the presence of the selection marker kanamycin and drug efflux activity was assessed. The efflux-active phenotype was observed after 3 h (**Figure 6**, top), whereas no phenotypic change was observed in the control experiment (**Figure 6**, bottom).

A prominent feature of the femtoliter droplet array is the ability to access individual droplets. Using a micropipette, not only droplets but also the cells within the droplets can be collected (**Figures 3B,C**). Collected single cells divide multiple times after transfer to growth medium in a test tube. The plasmid in the divided cells can be extracted and used for subsequent gene analysis (Iino et al., 2012a). Culturing after cell collection can be omitted by amplifying the DNA by single-cell PCR (Ottesen et al., 2006). Considering the rapid detection (3 h) of the phenotypic change after genetic transformation, single-cell gene analysis would enable high-throughput screening.

## PERSPECTIVE

As described above, the femtoliter droplet array is useful for single-bacterial cell analysis. Single-cell analysis of persister bacteria could help elucidate the mechanism of persister appearance and the reversible switching dynamics between persister and sensitive cells. This single-cell drug efflux assay can be used to screen for pump inhibitors, which requires the testing of numerous compounds. Our method is a direct evaluation of efflux activity, it takes only 20-30 min, and its advantage over the conventional method, based on the shift in the minimal inhibitory concentration is evident. Furthermore, with the advantage of individual droplet accessibility, single persister cells and cells exhibiting the efflux-active phenotype can be easily collected and used for subsequent analysis. It should be possible to screen for genes encoding functional efflux pump systems with a plasmid library of cloned genomic fragments. We believe that our approach will aid in addressing the challenge of infectious diseases caused by bacteria that are multidrug tolerant and resistant.

## Conflict of Interest Statement

The authors declare that the research was conducted in the absence of any commercial or financial relationships that could be construed as a potential conflict of interest.

## References

[B1] Allison K. R. Brynildsen M. P. Collins J. J. (2011). Heterogeneous bacterial persisters and engineering approaches to eliminate them. *Curr. Opin. Microbiol.* 14 593–598 10.1016/j.mib.2011.09.002 21937262 PMC3196368

[B2] Balaban N. Q. (2011). Persistence: mechanisms for triggering and enhancing phenotypic variability. *Curr. Opin. Genet. Dev.* 21 768–775 10.1016/j.gde.2011.10.001 22051606

[B3] Balaban N. Q. Merrin J. Chait R. Kowalik L. Leibler S. (2004). Bacterial persistence as a phenotypic switch. *Science* 305 1622–1625 10.1126/science.1099390 15308767

[B4] Boedicker J. Q. Vincent M. E. Ismagilov R. F. (2009). Microfluidic confinement of single cells of bacteria in small volumes initiates high-density behavior of quorum sensing and growth and reveals its variability. *Angew. Chem. Int. Ed. Engl.* 48 5908–5911 10.1002/anie.200901550 19565587 PMC2748941

[B5] Cai L. Friedman N. Xie X. S. (2006). Stochastic protein expression in individual cells at the single molecule level. *Nature* 440 358–362 10.1038/nature04599 16541077

[B6] Fischbach M. A. Walsh C. T. (2009). Antibiotics for emerging pathogens. *Science* 325 1089–1093 10.1126/science.1176667 19713519 PMC2802854

[B7] Gerdes K. Maisonneuve E. (2012). Bacterial persistence and toxin-antitoxin loci. *Annu. Rev. Microbiol.* 66 103–123 10.1146/annurev-micro-092611-150159 22994490

[B8] Gupta K. Kim D. H. Ellison D. Smith C. Kundu A. Tuan J. (2010). Lab-on-a-chip devices as an emerging platform for stem cell biology. *Lab Chip* 10 2019–2031 10.1039/c004689b 20556297

[B9] Henrichfreise B. Wiegand I. Pfister W. Wiedemann B. (2007). Resistance mechanisms of multiresistant *pseudomonas aeruginosa* strains from Germany and correlation with Hypermutation del. *Antimicrob. Agents Chemother.* 51 4062–4070 10.1128/aac.0014s-07 17876002 PMC2151423

[B10] Hocquet D. Nordmann P. El Garch F. Cabanne L. Plesiat P. (2006). Involvement of the MexXY-OprM efflux system in emergence of cefepime resistance in clinical strains of *Pseudomonas aeruginosa*. *Antimicrob. Agents Chemother.* 50 1347–1351 10.1128/AAC.50.4.1347-1351.2006 16569851 PMC1426951

[B11] Hocquet D. Roussel-Delvallez M. Cavallo J. D. Plesiat P. (2007). MexAB-OprM- and MexXY-overproducing mutants are very prevalent among clinical strains of *Pseudomonas aeruginosa* with reduced susceptibility to ticarcillin. *Antimicrob. Agents Chemother.* 51 1582–1583 10.1128/AAC.01334-06 17220417 PMC1855456

[B12] Iino R. Hayama K. Amezawa H. Sakakihara S. Kim S. H. Matsumono Y. (2012a). A single-cell drug efflux assay in bacteria by using a directly accessible femtoliter droplet array. *Lab Chip* 12 3923–3929 10.1039/c2lc40394c 22814576

[B13] Iino R. Nishino K. Noji H. Yamaguchi A. Matsumoto Y. (2012b). A microfluidic device for simple and rapid evaluation of multidrug efflux pump inhibitors. *Front. Microbiol. *3:40. 10.3389/fmicb.2012.00040 PMC3274760 22347225

[B14] Kim S. H. Iwai S. Araki S. Sakakihara S. Iino R. Noji H. (2012). Large-scale femtoliter droplet array for digital counting of single biomolecules. *Lab Chip* 12 4986–4991 10.1039/c2lc40632b 22961607

[B15] Kint C. I. Verstraeten N. Fauvart M. Michiels J. (2012). New-found fundamentals of bacterial persistence. *Trends Microbiol.* 20 577–585 10.1016/j.tim.2012.08.009 22959615

[B16] Koronakis V. Sharff A. Koronakis E. Luisi B. Hughes C. (2000). Crystal structure of the bacterial membrane protein TolC central to multidrug efflux and protein export. *Nature* 405 914–919 10.1038/35016007 10879525

[B17] Leung V. Levesque C. M. (2012). A stress-inducible quorum-sensing peptide mediates the formation of persister cells with noninherited multidrug tolerance. *J. Bacteriol.* 194 2265–2674 10.1128/JB.06707-11 22366415 PMC3347057

[B18] Lewis K. (2010). Persister cells. *Annu. Rev. Microbiol.* 64 357–372 10.1146/annurev.micro.112408.134306 20528688

[B19] Li G. W. Xie X. S. (2011). Central dogma at the single-molecule level in living cells. *Nature* 475 308–315 10.1038/nature10315 21776076 PMC3600414

[B20] Lindstrom S. Andersson-Svahn H. (2010). Overview of single-cell analyses: microdevices and applications. *Lab Chip* 10 3363–3372 10.1039/c0lc00150c 20967379

[B21] Livermore D. M. (2002). Multiple mechanisms of antimicrobial resistance in *Pseudomonas aeruginosa*: our worst nightmare? *Clin.Infect. Dis.* 34 634–640 11823954 10.1086/338782

[B22] Matsumoto Y. Hayama K. Sakakihara S. Nishino K. Noji H. Iino R. (2011). Evaluation of multidrug efflux pump inhibitors by a new method using microfluidic channels. *PLoS ONE *6:e18547. 10.1371/journal.pone.0018547 PMC3075257 21533264

[B23] Moker N. Dean C. R. Tao J. (2010). *Pseudomonas aeruginosa* increases formation of multidrug-tolerant persister cells in response to quorum-sensing signaling molecules. *J. Bacteriol.* 192 1946–1955 10.1128/JB.01231-09 20097861 PMC2838031

[B24] Morita Y. Kimura N. Mima T. Mizushima T. Tsuchiya T. (2001). Roles of MexXY- and MexAB-multidrug efflux pumps in intrinsic multidrug resistance of *Pseudomonas aeruginosa* PAO1. *J. Gen. Appl. Microbiol.* 47 27–32 12483565 10.2323/jgam.47.27

[B25] Murakami S. Nakashima R. Yamashita E. Matsumoto T. Yamaguchi A. (2006). Crystal structures of a multidrug transporter reveal a functionally rotating mechanism. *Nature* 443 173–179 10.1038/nature05076 16915237

[B26] Nakashima R. Sakurai K. Yamasaki S. Hayashi K. Nagata C. Hoshino K. (2013). Structural basis for the inhibition of bacterial multidrug exporters. *Nature* 500 102–106 10.1038/nature12300 23812586

[B27] Nakashima R. Sakurai K. Yamasaki S. Nishino K. Yamaguchi A. (2011). Structures of the multidrug exporter AcrB reveal a proximal multisite drug-binding pocket. *Nature* 480 565–569 10.1038/nature10641 22121023

[B28] Nikaido H. (2009). Multidrug resistance in bacteria. *Annu. Rev. Biochem.* 78 119–146 10.1146/annurev.biochem.78.082907.145923 19231985 PMC2839888

[B29] Nikaido H. Takatsuka Y. (2009). Mechanisms of RND multidrug efflux pumps. *Biochim. Biophys. Acta* 1794 769–781 10.1016/j.bbapap.2008.10.004 19026770 PMC2696896

[B30] Nishino K. Yamaguchi A. (2008). Role of xenobiotic transporters in bacterial drug resistance and virulence. *IUBMB Life* 60 569–574 10.1002/iub.90 18481812

[B31] Ottesen E. A. Hong J. W. Quake S. R. Leadbetter J. R. (2006). Microfluidic digital PCR enables multigene analysis of individual environmental bacteria. *Science* 314 1464–1467 10.1126/science.1131370 17138901

[B32] Pan J. Bahar A. A. Syed H. Ren D. (2012). Reverting antibiotic tolerance of *Pseudomonas aeruginosa* PAO1 persister cells by (Z)-4-bromo-5-(bromomethylene)-3-methylfuran-2(5H)-one. *PLoS ONE *7:e45778. 10.1371/journal.pone.0045778 PMC3447867 23029239

[B33] Pan J. Song F. Ren D. (2013). Controlling persister cells of *Pseudomonas aeruginosa* PDO300 by (Z)-4-bromo-5-(bromomethylene)- 3-methylfuran-2(5H)-one. *Bioorg. Med. Chem. Lett.* 23 4648–4651 10.1016/j.bmcl.2013.06.011 23810498

[B34] Rondelez Y. Tresset G. Tabata K. V. Arata H. Fujita H. Takeuchi S. (2005). Microfabricated arrays of femtoliter chambers allow single molecule enzymology. *Nat. Biotechnol.* 23 361–365 10.1038/nbt1072 15723045

[B35] Sakakihara S. Araki S. Iino R. Noji H. (2010). A single-molecule enzymatic assay in a directly accessible femtoliter droplet array. *Lab Chip* 10 3355–3362 10.1039/c0lc00062k 21031171

[B36] Sims C. E. Allbritton N. L. (2007). Analysis of single mammalian cells on-chip. *Lab Chip* 7 423–440 10.1039/b615235j 17389958

[B37] Taubes G. (2008). The bacteria fight back. *Science* 321 356–361 10.1126/science.321.5887.356 18635788

[B38] Teng S. W. Mukherji S. Moffitt J. R. de Buyl S O’Shea E. K. (2013). Robust circadian oscillations in growing cyanobacteria require transcriptional feedback. *Science* 340 737–740 10.1126/science.1230996 23661759 PMC3696982

[B39] Trouillon R. Passarelli M. K. Wang J. Kurczy M. E. Ewing A. G. (2013). Chemical analysis of single cells. *Anal. Chem.* 85 522–542 10.1021/ac303290s 23151043

[B40] Vega N. M. Allison K. R. Khalil A. S. Collins J. J. (2012). Signaling-mediated bacterial persister formation. *Nat. Chem. Biol.* 8 431–433 10.1038/nchembio.915 22426114 PMC3329571

[B41] Wakamoto Y. Dhar N. Chait R. Schneider K. Signorino-Gelo F. Leibler S. (2013). Dynamic persistence of antibiotic-stressed mycobacteria. *Science* 339 91–95 10.1126/science.1229858 23288538

[B42] Weibel D. B. Diluzio W. R. Whitesides G. M. (2007). Microfabrication meets microbiology. *Nat. Rev. Microbiol.* 5 209–218 10.1038/nrmicro1616 17304250

[B43] Yoshida K. Nakayama K. Ohtsuka M. Kuru N. Yokomizo Y. Sakamoto A. (2007). MexAB-OprM specific efflux pump inhibitors in *Pseudomonas aeruginosa*. Part 7: highly soluble and in vivo active quaternary ammonium analogue D13-9001, a potential preclinical candidate. *Bioorg. Med. Chem.* 15 7087–7097 10.1016/j.bmc.2007.07.039 17869116

[B44] Zgurskaya H. I. Nikaido H. (1999). AcrA is a highly asymmetric protein capable of spanning the periplasm. *J. Mol. Biol*. 285 409–420 10.1006/jmbi.1998.2313 9878415

